# AFTER A NEUROCOGNITIVE EVALUATION: ROLES AND COMPETENCIES OF BEHAVIORAL HEALTH PROVIDERS

**DOI:** 10.1093/geroni/igac059.794

**Published:** 2022-12-20

**Authors:** Rebecca Allen, Ann Steffen

**Affiliations:** The University of Alabama, TUSCALOOSA, Alabama, United States; University of Missouri-St. Louis, St. Louis, Missouri, United States

## Abstract

GSA’s revised KAER Toolkit for Primary Care Teams (Fall, 2020) provides useful information for referring individuals and families to community resources following a neurocognitive disorder diagnosis. Mental health practitioners must develop competencies and professional identities within integrated care teams to facilitate this process. This presentation outlines post-assessment suggestions for clinicians across disciplines (psychology, social work, nursing, medicine, pharmacy, +) in building such teams. There may be needs for serial assessment of specific individual capacities, treatment plans to facilitate shared decision-making between individuals and family care partners, and evaluations of the family care partner’s capacity to provide care. Behavioral health providers assist patients, families, and health care teams across a range of residential settings in understanding and implementing treatment recommendations. Emerging science indicates the value of initiating planning discussions with lifestyle modifications to enhance functioning within dyads. Culturally responsive strategies will be suggested for engagement in planning for future care.

